# Perceived Multisensory Common Cause Relations Shape the Ventriloquism Effect but Only Marginally the Trial‐Wise Aftereffect

**DOI:** 10.1111/ejn.70015

**Published:** 2025-02-11

**Authors:** Christoph Kayser, Herbert Heuer

**Affiliations:** ^1^ Department of Cognitive Neuroscience Universität Bielefeld Bielefeld Germany; ^2^ Leibniz Research Centre for Working Environment and Human Factors Dortmund Germany

**Keywords:** audio‐visual, cross‐modal, multisensory, multisensory causal inference

## Abstract

Combining multisensory cues is fundamental for perception and action and reflected by two frequently studied phenomena: multisensory integration and sensory recalibration. In the context of audio‐visual spatial signals, these phenomena are exemplified by the ventriloquism effect and its aftereffect. The ventriloquism effect occurs when the perceived location of a sound is biased by a concurrent visual stimulus, while the aftereffect manifests as a recalibration of perceived sound location after exposure to spatially discrepant stimuli. The relationship between these processes—whether recalibration is a direct consequence of integration or operates independently—remains debated. We investigate the role of causal inference in these processes by examining whether trial‐wise judgements about a common‐cause underlying audio‐visual stimuli influence both the ventriloquism effect and the immediate aftereffect. In a spatial paradigm, participants made explicit judgements about the common cause of stimulus pairs, and their influence on both perceptual biases was assessed. Results obtained across two experiments indicate that while multisensory integration is contingent on common cause judgements, the immediate recalibration effect is not. This suggests that recalibration can occur independently of the perceived commonality of the multisensory stimuli, challenging the notion that recalibration is solely a by‐product of integration.

## Graphical abstract

The ventriloquism effect describes how simultaneously presented visual and acoustic signals are combined during perception. This effect depends on whether the two stimuli are judged as arising from a common cause. We here asked whether the ventriloquism aftereffect, which describes the biased localization of a subsequent sound, also depends on the preceding multisensory stimuli to be perceived as originating from a common cause.

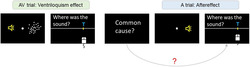

**Abbreviations**
BF: Bayes factor
ccj: common‐cause judgement

## 1 Introduction

Combining multiple sensory cues is central to perception and action during daily activities. Current research differentiates two kinds of such multisensory combinations: the integration of discrepant signals, as revealed by perceptual judgements of multisensory stimuli, and the recalibration of one or both signals, as revealed by the aftereffect of discrepant stimuli on subsequent judgements of unisensory stimuli. In the context of audio‐visual spatial perception, these multisensory combinations are exemplified by the ventriloquism effect and aftereffect (Bertelson and Radeau 1981; Recanzone 1998). The ventriloquism effect reflects how our judgements of (e.g.) a sound's location are biased by seeing a visual stimulus at the same time. The aftereffect reflects how exposure to spatially discrepant audio‐visual stimuli biases the localization of subsequently presented sounds, cumulatively with repeated exposures. The relation between the ventriloquism effect and aftereffect, and thus the underlying processes of integration and recalibration, is still debated.

One line of studies supports that the aftereffect is a direct consequence of the preceding integration of multisensory signals (Bruns 2019; Rohlf et al. 2020; Noppeney 2021; Rohlf, Bruns, and Röder 2021). Both integration and recalibration scale similarly with the discrepancy of the multisensory stimuli, this discrepancy may directly drive both processes (Wozny and Shams 2011b; Bruns and Roder 2015; Park and Kayser 2019). Furthermore, both are similarly affected by manipulations of stimulus reliability (Rohlf, Bruns, and Röder 2021) and attention (Badde, Navarro, and Landy 2020), and neuroimaging studies point to partly overlapping neurophysiological processes shaping both (Park and Kayser 2019, 2021). It is known that attributing a common cause to multisensory stimuli is prerequisite to their features being integrated (Ernst and Bulthoff 2004; Wallace et al. 2004; Kording et al. 2007; Sato, Toyoizumi, and Aihara 2007). Indeed, the perceptual bias arising from integration emerges only in trials in which the two stimuli are judged as being causally related (Rohe and Noppeney 2015). If recalibration was a direct consequence of integration, recalibration should also depend on whether the two spatially discrepant stimuli are judged as originating from a common source. However, it remains unclear whether this is indeed the case.

Another line of studies argues that recalibration is driven mainly by a belief in a modality‐specific bias. In the case of the ventriloquism aftereffect, this would be a bias specific to estimates of acoustic features that should emerge independently of whether the preceding multisensory signals have been integrated or not (Di Luca, Machulla, and Ernst 2009; Block and Bastian 2011; Zaidel, Ma, and Angelaki 2013; Bosen et al. 2017). If this notion was correct, recalibration should emerge largely independently of whether the multisensory stimuli are judged as being causally related, that is, originating from a common source.

These predictions should be particularly true on a trial‐by‐trial level. Recalibration is observed following a single trial of audio‐visual exposure (known as immediate or trial‐wise aftereffect), but also growths cumulatively with extended exposure. Hence, recalibration depends on both the sensory information received during an individual trial as well as the consistency of this over an extended period (Bruns and Roder 2015; Bosen et al. 2017). Indeed, when probed across sequences of multisensory trials, integration depends only on the currently presented spatial discrepancy while recalibration is sensitive to the series of preceding discrepancies (Kayser, Park, and Heuer 2023). The different time scales on which integration and recalibration operate could come along with different time scales of dependence on multisensory common cause evidence (Debats, Heuer, and Kayser 2023a). Thus, whereas, on a given trial, multisensory integration emerges only for a pair of discrepant multisensory stimuli attributed to a common cause in that trial, immediate recalibration following these stimuli may only minimally depend on this short‐term common cause evidence.

The present study was designed to arbitrate between these alternatives by probing the influence of trial‐wise common cause judgements on the ventriloquism effect and the immediate aftereffect in an audio‐visual spatial paradigm. This paradigm has been used previously to study these phenomena in different contexts (Park and Kayser 2019, 2020, 2022; Kayser, Park, and Heuer 2023). We here expanded this paradigm by including explicit trial‐wise judgements about participants' belief that audio‐visual stimulus pairs were originating from a common cause or not and tested the predictive power of these common cause judgements on both biases in two experiments, one replicating the other. Specifically, we focus on the participant‐wise dependency of each bias on the spatial discrepancy and ask whether these dependencies are shaped by the common‐cause judgements. Our results show that the integration of discrepant stimuli depends on whether they are judged as originating from a common cause, as expected, while the immediate ventriloquism aftereffect does not.

## 2 Methods

### 2.1 Participants

We report data from two experiments in which 44 adult volunteers participated after providing informed consent. All had self‐reported normal vision and hearing, and none indicated a history of neurological disorders. The procedures were approved by the ethics committee of Bielefeld University, and the data were collected anonymously. Participants were compensated financially for their time. For experiment 1, we collected data from 23 participants and for experiment 2 from 21 participants. Experiment 2 was implemented to reproduce the results obtained in experiment 1 using a different type of common cause response method, as explained below. These sample sizes are in line with previous studies using similar experimental protocols (Park and Kayser 2019; Park, Nannt, and Kayser 2021; Park and Kayser 2022) and recommendations for behavioural studies (Simmons, Nelson, and Simonsohn 2011).

### 2.2 Experimental Setup and Paradigm

The experiments were based on an established single‐trial audio‐visual localization task in which the individual trials either probe the ventriloquism effect or the immediate ventriloquism aftereffect (Wozny and Shams 2011b; Park and Kayser 2019, 2021, 2022). They were conducted in an echo‐free booth (E:Box, Desone) where participants sat in front of an acoustically transparent screen (Screen International Modigliani, 2 × 1 m^2^, about 1 m from the participant's head) with their head supported on a chin rest. The experiments comprised audio‐visual (AV) trials and auditory (A) trials and less frequent visual (V) trials. These visual trials were mainly included to maintain attention divided across modalities. Participants' task was to localize either the sound (in AV or A trials) or the visual stimulus (in V trials). They were asked to fixate a central dot during the pre‐stimulus and stimulus periods but could move their eyes during responding and inter‐trial intervals.

Each trial started with a fixation period (uniform 700–1100 ms), followed by stimulus presentation (50 ms). After a post‐stimulus interval (uniform 400–700 ms), participants had to indicate the position of the auditory (in AV and A trials) or visual (in V trials) stimulus. A horizontal bar was presented along which participants could move a mouse cursor to indicate their judgement by pressing the mouse button. In AV trials, participants were subsequently asked (after a uniform interval of 200–300 ms) to indicate whether they perceived the auditory and visual stimuli as ‘to arise from the same source’ or ‘from different sources’ (common cause judgement). In experiment 1, participants were instructed to move a mouse cursor along a vertical bar ‘to a position that would mark the strength of their belief’ about common or distinct causes; the two extreme positions were labelled ‘common cause’ (highest position) and ‘separate causes’ (lowest position).

After running experiment 1, we realized that many participants tended to move the mouse cursor to the extreme positions and effectively submitted binary decisions. Possibly because it was not explicitly clear for participants how to translate the given instruction (‘Indicate whether you believe these two stimuli arise from the same/different sources’) into a continuous response, and many participants may in fact have used a binary decision criterion. For this reason, we implemented experiment 2, in which the common cause judgement was directly performed as a binary decision, with any movements of the mouse cursor upwards from the midline labelled as ‘common cause’ and those downwards as ‘separate causes’. We still relied on the use of a mouse cursor for this binary common‐cause judgement (rather than, e.g., a keyboard) given that the spatial localization response necessary relied on the same response modality. In both experiments, participants finalized the common cause judgement by clicking a mouse button. Trials were separated by inter‐trial intervals between 800 and 1200 ms.

Stimulus presentation was controlled using the Psychophysics toolbox (Brainard 1997) for MATLAB (The MathWorks Inc., Natick, MA) with confirmed temporal synchronization of auditory and visual stimuli. The acoustic stimuli were presented through speakers (Monacor MKS‐26/SW, MONACOR International GmbH & Co. KG) that were located behind the visual screen at 5 discrete horizontal locations (±22°, ±11°, 0°), with the centre position always being directly in front of the participant. Sound presentation was controlled via a multi‐channel soundcard (Creative Sound Blaster Z) and amplified via an audio amplifier (t.amp E4‐130, Thomann). The acoustic stimulus was a 1300‐Hz sine wave tone (50‐ms duration; sampled at 48 kHz) that was presented at one of two signal‐to‐noise ratios. These were introduced to manipulate the reliability of the acoustic spatial information and thereby the integration bias and possibly the common cause judgements. In the high reliability condition (labelled A+), the tone at the target position was presented at 65‐dB SPL, while the same tone was presented from all other four speakers at ~45‐dB SPL. In the low reliability condition (labelled A−), the target was presented at 58 dB, hence with less intensity difference to the other speakers. Visual stimuli were clouds of dots distributed according to a two‐dimensional Gaussian distribution (200 dots, SD of vertical and horizontal spread = 2°, width of a single dot = 0.12°, duration 50 ms) similar to previous studies (Park and Kayser 2019, 2021). They were projected (Acer Predator Z650, Acer Inc., Taiwan) onto the acoustically transparent screen.

Experiment 1 featured a total of 832 trials administered in 6 blocks (of these 360 AV trials, 360 A trials and 112 visual trials). Auditory and visual stimuli were presented at the same or different discrete locations, inducing a multisensory spatial discrepancy that ranged from −44° to +44° in steps of 11°. The specific locations of auditory and visual stimuli within AV trials were sampled randomly from a distribution that was constructed such as to present each spatial discrepancy with equal frequency. To this end, the unisensory stimulus positions with intermediate eccentricity (±11°) were overrepresented compared to the other locations. The order of the individual positions and discrepancies was randomized across trials. The locations of stimuli in the A (and also separately for V trials) were sampled from a uniform distribution across the five potential locations and presented in random order across trials. Also, the positions of the sounds in the AV and subsequent A trials were sampled independently. The responses to the largest discrepancies of ±44 proved rather variable. Therefore, this discrepancy was not included in the design of experiment 2, which was otherwise similar. Experiment 2 featured a total of 742 trials administered in 4 blocks (of these 306 AV trials 306, A trials and 126 visual trials), with discrepancies ranging from −33° to + 33°.

One or two blocks of practice trials were run prior to the main experiment to familiarize participants with the task and stimuli. During these trials, feedback was provided by dividing the judgement error into three classes and providing respective feedback (‘good’, ‘intermediate’ and ‘off’). Before the actual experiment, participants were also screened for their spatial hearing abilities. They were asked to localize noise bursts (50‐ms duration, 65‐dB SPL) presented from the 4 lateralized speakers in a left/right two‐choice task, performing 10 repeats per location. All participants performed above an average of 75% correct responses.

### 2.3 Definition of Trial‐Wise Judgement Errors

The trial‐wise judgement errors were defined so as to take potentially confounding response biases into account, such as a bias towards the midline of the screen (Kording et al. 2007; Wozny and Shams 2011a, 2011b; Park and Kayser 2019). Practically, we computed the deviation between the participant's trial‐wise response and the expected response given the location of the judged sound in that trial, similar to previous work. This expected response was computed for each sound location as the average response across all trials with a sound at this location. In line with previous studies (Kayser, Park, and Heuer 2023; Kayser, Debats, and Heuer 2024), we removed individual trials with judgement errors larger than 150% of the largest spatial discrepancy included in each experiment. These trials can reflect lapses of attention or accidental clicks when submitting the response. From experiment 1, we also removed trials in which the spatial discrepancy was ±44°, as these responses exhibited a much larger trial‐to‐trial variability than those at other discrepancies.

### 2.4 Analysis of Common Cause Judgements

**FIGURE 1:**
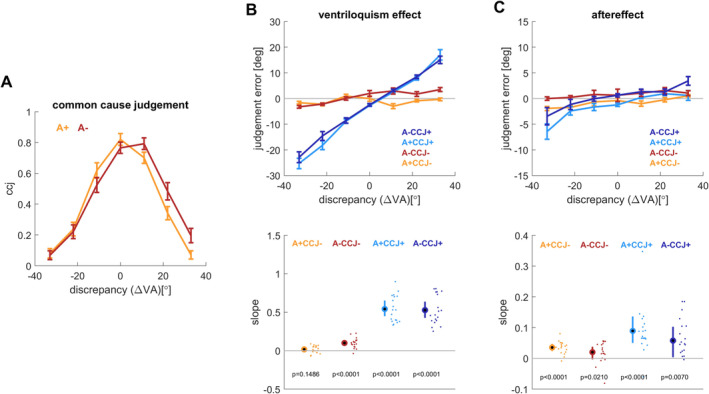
Results for experiment 1 (*n* = 19). (A) Common cause judgements as a function of the spatial discrepancy and auditory reliability (high: A+, low: A−). (B) Ventriloquism effect and (C) aftereffect. Both are shown as the respective continuous judgement errors for each of the conditions (upper panel) and as participant‐wise slopes against the spatial discrepancy (lower panel). These slopes were derived by fitting the models to the condition‐ and participant‐wise data (Equation 1). The indicated *p*‐values reflect a test of the group‐level mean against zero (paired *t*‐tests). Lines indicate means and standard errors, dots individual participants. CCJ+: trials on which participants rated the audio‐visual stimuli as to arise from a common cause; CCJ−: trials on which participants rated them as to arise from separate sources. Note the different scales on the ordinates for ventriloquism effect and aftereffect.

**FIGURE 2:**
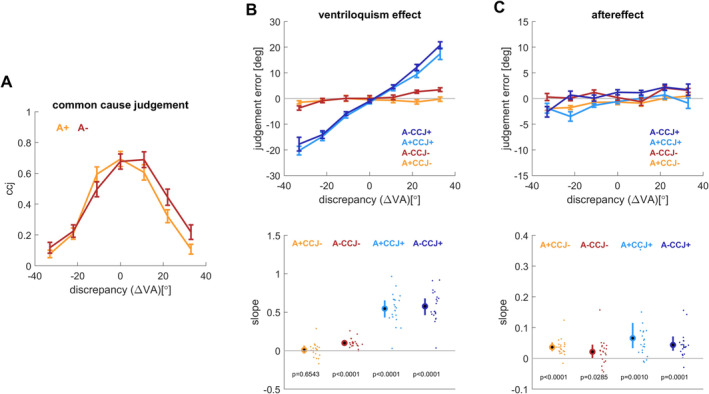
Results for experiment 2 (*n* = 21). (A) Common cause judgements as a function of the spatial discrepancy and auditory reliability (high: A+, low: A−). (B) Ventriloquism effect and (C) aftereffect. Both are shown as the respective continuous judgement errors for each of the conditions (upper panel) and as participant‐wise slopes against the spatial discrepancy (lower panel). These slopes were derived by fitting the models to the condition‐ and participant‐wise data. The indicated *p*‐values reflect a test of the group‐level mean against zero (paired *t*‐tests). Lines indicate means and standard errors, dots individual participants. CCJ+: trials on which participants rated the audio‐visual stimuli as to arise from a common cause; CCJ−: trials on which participants rated them as to arise from separate sources. Note the different scales on the ordinates for ventriloquism effect and aftereffect.

In experiment 1, common cause judgements were provided on a continuous scale, but participants often used only the extremes of the continuum. Hence, we converted the common cause judgements to a binary variable for all participants. To account for potential individual biases in how participants used the continuous scale, we split the judgements of each participant by the overall arithmetic mean judgement for this participant. Note that we used the mean and not the median to split trials, as for some participants, the median was one of the two extreme values. Still, for 4 of the 23 participants, this left not sufficient variability in the common cause judgements, and these participants were excluded. In experiment 2, participants' responses were directly saved as a binary response for each trial and were used as such for analysis. Comparing the common cause judgements across experiments confirms that these are very similar (Figures 1 and 2), and overall, the results from both experiments conform very well.

### 2.5 Analysis of the Ventriloquism Bias and Aftereffect

The ventriloquism bias was defined based on the judgement errors in the AV trials, and the aftereffect was defined based on the judgement errors in the A trials. These judgement errors scale with the audio‐visual spatial discrepancy ΔVA, defined as the location of the visual stimulus minus that of the sound in the AV trials. We characterized the strength of each bias separately for each experimental condition (defined by the auditory reliability, high or low and the common‐cause judgement, same or different) as the slope of the trial‐wise judgement error against the trial‐wise spatial discrepancy. We obtained this slope from the following regression:

(1) $$\text{Judgement error}\sim b^{*}\;\mathrm{\Delta VA}+\text{offset}$$

These slopes were computed for each participant and condition and are shown in the bottom panels of Figures 1 and 2.

While previous work shows that this provides a good description of both the ventriloquism and the aftereffect, it is known that the ventriloquism bias can also scale in a non‐linear manner with spatial discrepancy. This non‐linear dependency describes the reduced tendency to bind multisensory stimuli when these are seemingly discrepant and not judged as arising from a common cause (Kording et al. 2007; Rohe and Noppeney 2015; Shams and Beierholm 2022), following Bayesian models of sensory causal inference. However, as we here fit the judgement errors separately for trials in which participants judged the two stimuli as originating from one or two separate causes, a linear model provides an adequate description of the data. To verify this, we also fit the judgement errors for the ventriloquism bias with a model comprising both the linear term (as in Equation 1) and a non‐linear term, the signed square‐root of the magnitude of the discrepancy (see, e.g., Park and Kayser 2020, 2022). Across the data from all 40 participants in both experiments, and averaged across the four conditions, the explained variance was essentially identical for the linear model and the combined linear/non‐linear model (*R* ^2^ linear: 0.41 ± 0.02; *R* ^2^ linear + quadratic: 0.42 ± 0.02, paired *t*‐test *p* = 0.45, *t* = 0.75). These condition‐ and participant‐wise slopes served as the main characteristics of interest on which we based the statistical tests of our main questions.

### 2.6 Statistical Hypothesis Testing

Overall, we report results for 19 participants in experiment 1, based on 275 ± 16 AV/A trial‐pairs (mean ± SD) and results for 21 participants in experiment 2, based on 303 ± 13 AV/A trial‐pairs. The main questions concerned the role of the common‐cause judgement (ccj) in shaping the ventriloquism bias and the aftereffect. We tested for an effect of ccj together with an effect of the reliability (Rel) of the acoustic stimuli using linear mixed‐effects models. These were applied to each experiment and bias separately. Specifically, we modelled the bias (slopes) based on both effects and their interaction, while also allowing for random effects of individual participants (S):

(2) $$\text{Bias}\;b\sim \mathrm{Rel}+\mathrm{ccj}+\mathrm{Rel}:\mathrm{ccj}+\left(1|S\right)+\left(\mathrm{Rel}|S\right)+\left(\mathrm{ccj}|S\right)$$

The terms in brackets indicate random offsets and slopes for individual participants. Models were fit using a maximum likelihood procedure in Matlab R2021a (fitglme.m). From these models, we extracted the statistical significance of each fixed factor (Tables 2 and 3). In addition, we compared the predictive power offered by either the common‐cause judgement or the reliability by comparing the model in Equation (2) with a reduced model omitting the specific factor of interest (e.g., omitting all terms including ccj). We compared this predictive power based on the Bayesian information criteria (BIC) associated with each model. To judge differences in predictive power, we relied on a Bayesian approach and converted differences in BICs to Bayes factors, as follows (Wagenmakers 2007; Wagenmakers et al. 2018): BF = exp((BIC1 − BIC0)/2). Bayes factors in favour of no effect of a predictor are reported as negative numbers, rather than as fractions smaller than one (i.e., Bayes factors smaller than 1 were converted as −1/BF). When interpreting Bayes factors, we refer to the nomenclature of Raftery (1995) and interpret BF between 1 and 3 as ‘weak’, between 3 and 20 as ‘positive’, between 20 and 150 as ‘strong’ and BF > 150 as ‘very strong’ evidence.

Besides the main question concerning the role of the common‐cause judgement in shaping the biases, we also quantified the dependency of the ccj itself on the spatial discrepancy and auditory reliability. We modelled the trial‐wise (binary) common cause judgement based on the auditory reliability, the magnitude of the discrepancy and their interaction using a binomial model with a logistic link function:

(3) $$\mathrm{ccj}\sim \mathrm{\Delta VA}+\mathrm{Rel}:\mathrm{\Delta VA}+\mathrm{Rel}+\left(1|S\right)+\left(\mathrm{Rel}|S\right)$$

Again, we compared the BIC value of models including or omitting a factor of interest (e.g., Rel) to provide evidence concerning the predictive power of this on the common cause judgement.

## 3 Results

### 3.1 Common Cause Judgements

The common cause judgements (ccj) scaled with the audio‐visual discrepancy and participants judged two stimuli as less likely to emerge from a common cause when separated by a larger distance (Figures 1A and 2A). For statistical analysis, we modelled the trial‐wise ccj against the magnitude of the spatial discrepancy, the reliability of the auditory stimuli and their interaction (Table 1). For both experiments, this revealed very strong evidence in favour of a role of the spatial discrepancy in predicting the ccj and also evidence in favour of a role of the auditory reliability (Table 1). This dependency of the ccj on the spatial discrepancy confirms previous studies using a similar paradigm (Rohe and Noppeney 2015).

**TABLE 1:** Influence of auditory reliability and multisensory discrepancy on common cause judgements.

	Beta ± SD	*t*	*p*	BF
Experiment 1				
Intercept	2.453 + 0.277	8.857	< 0.0001	
|ΔVA|	−0.169 + 0.012	−14.378	< 0.0001	10^10^
Rel	−0.356 + 0.141	−2.527	0.0115	3.6
Rel:|ΔV|	0.028 + 0.007	3.966	< 0.0001	
Experiment 2				
Intercept	1.733 + 0.246	7.030	< 0.0001	
|ΔVA|	−0.143 + 0.010	−14.860	< 0.0001	10^10^
Rel	−0.288 + 0.117	−2.466	0.0137	10^3^
Rel:|ΔV|	0.029 + 0.006	4.924	< 0.0001	

*Note:* For each experiment, we modelled the trial‐wise common cause judgement against the magnitude of the spatial discrepancy (|Δva|), the reliability of the acoustic signal (levels high reliability, low reliability) and their interaction. The table lists the respective coefficients of each factor including their standard deviation, the respective *t*‐values and *p*‐values. The Bayes factor quantifies the relevance of each factor, obtained by contrasting models with and without the factor of interest (both as isolated term and in interactions).

The data in Figures 1 and 2A also suggest that there is a slight spatial shift in the common cause judgement with the reliability of the acoustic stimuli. We cannot directly explain this and cannot rule out that a potential calibration issue with one of the speakers contributes to this. However, it seems unlikely that this shift confounds with the main questions investigated here, of whether the common cause judgement has different impact on the ventriloquism effect and the aftereffect.

### 3.2 Ventriloquism Effect

As expected based on previous work, the judgement errors reflecting the ventriloquism bias scaled with the spatial discrepancy. The upper panels in Figures 1 and 2B show the scaling of the condition‐wise bias with discrepancy, the lower panels the participant‐wise slopes of the individual judgement errors against discrepancy. These slopes serve as the main variable to test the question of interest. These condition‐wise slopes were significantly different from zero when participants judged the audio‐visual stimuli as originating from a common cause, both with the high and low auditory reliability, but not in the condition of no‐common cause judgement and high auditory reliability (paired *t*‐tests; see insets in Figures 1 and 2B).

To directly probe for an effect of ccj and reliability, we tested the predictive power of both factors on the participant‐wise slopes (Table 2). While reliability was a significant predictor for the slope in both experiments, the added predictive power of this factor (over ignoring this factor) was small; a Bayesian model comparison offered positive evidence against an effect of reliability (Bayes factors in Table 2). In contrast, the ccj was a highly significant predictor and the Bayes factors provide very strong evidence for the explanatory power of this variable. Note that the interactions of reliability with ccj were also significant.

**TABLE 2:** Influence of auditory reliability and common cause judgements on the ventriloquism effect.

	Beta ± SD	*t*	*p*	BF
Experiment 1				
Intercept	−0.690 + 0.071	−9.734	< 0.0001	
Rel	0.181 + 0.040	4.491	< 0.0001	−6.2
ccj	0.623 + 0.053	11.862	< 0.0001	> 10^5^
Rel:ccj	−0.098 + 0.025	−3.903	0.0002	
Experiment 2				
Intercept	−0.679 + 0.078	−8.690	< 0.0001	
Rel	0.151 + 0.043	3.485	0.0008	−4.1
ccj	0.598 + 0.058	10.307	< 0.0001	> 10^5^
Rel:ccj	−0.060 + 0.027	−2.206	0.0302	

*Note:* For each experiment, we modelled the condition‐ and participant‐wise slopes of the ventriloquism bias against auditory reliability (Rel), the common cause judgement (ccj) and their interaction as explained in Section 2 (Equation 2). The table lists the respective coefficients of each factor including their standard deviation, the respective *t*‐values and *p*‐values. The Bayes factor quantifies the relevance of each factor, obtained by contrasting models with and without the factor of interest (both as isolated term and in interactions).

### 3.3 Aftereffect

The aftereffects in the two experiments were robust and reflect the spatial scaling of the judgement error with the spatial discrepancy in the preceding auditory trial. The upper panels in Figures 1 and 2B show the scaling of the aftereffect with discrepancy, the lower panels show the participant‐wise slopes. Consistent with previous work (Park and Kayser 2021, 2022), the slopes of the aftereffect were smaller than those of the ventriloquism effect, but they were consistently positive and significantly different from zero (see inset in Figures 1 and 2B).

The statistical analysis provided positive to very strong evidence against an influence of auditory reliability, and neither the predictor reliability nor its interaction with the ccj were significant (Table 3). Importantly, also the common‐cause as predictor was not significant for either experiment, and the models suggested positive and very strong evidence against a predictive benefit of the ccj in explaining the aftereffect (Bayes factors in Table 3).

**TABLE 3:** Influence of auditory reliability and common cause judgements on the aftereffect.

	Beta ± SD	*F*	*p*	BF
Experiment 1				
Intercept	−0.020 + 0.056	−0.350	0.7276	
Rel	0.001 + 0.035	0.030	0.9760	−3561
CCJ	0.070 + 0.036	1.970	0.0527	−8.2
Rel:CCJ	−0.016 + 0.022	−0.748	0.4566	
Experiment 2				
Intercept	0.015 + 0.048	0.309	0.7582	
Rel	−0.008 + 0.030	−0.267	0.7899	−460
CCJ	0.036 + 0.029	1.269	0.2082	−1328
Rel:CCJ	−0.007 + 0.018	−0.388	0.6994	

*Note:* For each experiment, we modelled the condition‐ and participant‐wise slopes against auditory reliability (Rel), the common cause judgement (ccj) and their interaction as explained in the Methods (Equation 2). The table lists the respective coefficients of each factor including their standard deviation, the respective *t*‐values and *p*‐values. The Bayes factor quantifies the relevance of each factor, obtained by contrasting models with and without the factor of interest (both as isolated term and in interactions).

## 4 Discussion

Multisensory integration and recalibration are two processes by which the brain deals with seemingly discrepant sensory signals. Though both are well investigated, their relation remains a matter of discussion. Multisensory integration supposedly reduces the discrepancy between two redundant sensory signals that are received more or less at the same time and judged as originating from a common cause (Kording et al. 2007; Rohe and Noppeney 2015; Zierul et al. 2019; Badde, Navarro, and Landy 2020; Shams and Beierholm 2022). Recalibration, in contrast, supposedly serves to reduce persistent or constant discrepancies between sensory estimates and begins to emerge after a single exposure to discrepant audio‐visual stimuli (Bruns and Roder 2015; Bosen et al. 2017; Bosen et al. 2018). According to one hypothesis, recalibration is driven by the belief in a modality specific bias and not strictly tied to the integration of the multisensory stimuli driving recalibration (Block and Bastian 2011; Zaidel, Ma, and Angelaki 2013; Bosen et al. 2017; Bruns and Röder 2019; Tong et al. 2020). We here support this idea and show that the trial‐wise belief that two spatially discrepant multisensory stimuli originate from a common cause is a prerequisite for integration, but the immediate aftereffect shows no dependence on it. This would explain why the aftereffect can also emerge when auditory and visual signals obviously do not originate from a common location (Radeau and Bertelson 1974) and when attention is directed towards task‐unrelated stimuli (Eramudugolla et al. 2011).

### 4.1 Trial‐Wise Recalibration Does Not Depend on Trial‐Wise Common Cause Judgements

In the type of paradigm used here, the trial‐wise aftereffect is statistically correlated with both the spatial discrepancy in the preceding multisensory trial and participants' trial‐wise integration bias (Park and Kayser 2019, 2021). However, this does not imply that the trial‐wise integration causally drives recalibration. In fact, based on the analysis of EEG data, we have concluded that the neurophysiological drivers of the trial‐wise aftereffect are more shaped by the discrepancy than by the integration bias observed in the multisensory trials (Park and Kayser 2021). Hence, while both the sensory signals and their resulting interpretation and action in a multisensory trial may feed into recalibration (Park and Kayser 2021; Park, Nannt, and Kayser 2021), the sensory signals are what directly shapes the trial‐wise aftereffect. Based on this results on may predict that while integration is contingent on attributing both stimuli to a common cause, the trial‐wise aftereffect should not. This is just what we observed here, consistently across two experiments probing the same effect in similar but not identical paradigms. Based on Bayesian model comparisons, the data provide very strong evidence in favour of a predictive effect of the common‐cause judgement on the ventriloquism bias and positive to very strong evidence against a predictive effect on the aftereffect.

In line with previous work, the current results support the notion that recalibration is shaped by the belief in a modality‐specific bias, here one pertaining to the auditory system. Multiple factors may shape this belief, some of which also shape integration; hence, both biases often tend to correlate across experimental manipulations. However, these biases show a differential sensitivity to the history of the multisensory signals. When probed across sequences of experimental trials, integration depends only on the discrepancy of the audio‐visual signals in the current trial, while recalibration reflects both the immediate and preceding past (Kayser, Park, and Heuer 2023). A similar history dependence may also pertain to the common cause evidence, where integration depends mostly on the momentary common cause evidence while recalibration depends on the common‐cause evidence as observed over a longer time frame. Indeed, in a visuo‐motor paradigm, we have shown that reducing the inter‐trial evidence for a common cause reduces integration but not recalibration (Debats, Ernst, and Heuer 2017; Debats, Heuer, and Kayser 2023b). Hence, recalibration may be more sensitive to the evidence for sensory‐causal relations on a longer time scale, while integration is contingent on the immediate common cause evidence. However, future work is required to directly probe to which degree either integration or recalibration scale with the experienced history of a number of sequent common‐cause judgements. Given that such an analysis requires a substantial amount of data to properly sample the history of previous trials (Kayser, Park, and Heuer 2023), the present data do not offer a direct opportunity to test this.

This dependency of integration and recalibration on prior stimuli or actions is also of interest in the broader context of sequential effects in perceptual decision making. A previous study has suggested that some of the classical reaction time benefits seen in uni‐/multi‐sensory detection paradigms can be attributed to amodal switching costs in cognition (Shaw et al. 2020). This raises the general question to what degree multisensory perceptual phenomena arise from the genuine integration of multiple signals and to what degree amodal processes related to parallel or sequential processing, task flexibility and the allocation of attention contribute to these. Together with the general context‐ and history‐dependence of these effects (Beierholm et al. 2020; Park and Kayser 2022), this raises many interesting questions for future work.

### 4.2 Integration and Recalibration From a Bayesian Perspective

We interpret the collective results obtained previously (Park and Kayser 2021, 2022; Debats, Heuer, and Kayser 2023b; Kayser, Park, and Heuer 2023) as follows: The combination of multisensory signals is generally shaped by models of Bayesian causal inference (Noppeney 2021; Shams and Beierholm 2022). Unpredictable discrepancies emerging for stimuli that are assumed to arise from a common cause are reduced on a trial‐by‐trial level through multisensory integration. At the same time, the brain continuously updates the a priori belief about a common cause of the multisensory signals based on the recently experienced discrepancies (Kording et al. 2007; Beierholm et al. 2020), attention (Badde, Navarro, and Landy 2020) and the overall context in which the sensory information is received. Separately, at least one of the consistently discrepant sensory signals (e.g., the representation of auditory space here) is continuously updated in a leaky manner to compensate for short‐term changes in audio‐visual disparities and to minimize apparent localization errors (Recanzone 1998; Kopco et al. 2009; Wozny and Shams 2011a; Bosen et al. 2018). The estimates of multisensory discrepancies that feed into this update may be indirectly shaped by the common cause evidence, but the data suggest that distinct time scales of this are relevant for integration and recalibration: For integration, the immediate common cause evidence is most relevant, while for recalibration, it is the long‐term evidence—and the immediate evidence may matter only as a potential start of long‐term evidence. Dissociating these time scales of common cause is difficult but not impossible, and it is a manipulation that holds promise to better dissociate the factors shaping integration and recalibration in future work.

## Author Contributions

Designed the study: C.K. and H.H.; collected data: C.K.; data analysis: C.K.; data interpretation: C.K. and H.H.; manuscript preparation: C.K. and H.H.

## Conflicts of Interest

The authors declare no conflicts of interest.

### Peer Review

The peer review history for this article is available at https://www.webofscience.com/api/gateway/wos/peer‐review/10.1111/ejn.70015.

## Data Availability

The data and relevant Matlab code to create the figures and main analyses are available under https://github.com/christophckayser/Kayseretal_EJN24_ccj.
